# Development of a Semi-Quantitative Food Frequency Questionnaire to Estimate Macronutrient Intake among Type 2 Diabetes Mellitus Patients in Malaysia

**DOI:** 10.3390/nu15030506

**Published:** 2023-01-18

**Authors:** Norizzati Amsah, Zaleha Md Isa, Norfazilah Ahmad

**Affiliations:** Department of Community Health, Faculty of Medicine, Universiti Kebangsaan Malaysia, Kuala Lumpur 56000, Malaysia

**Keywords:** development, food frequency questionnaire, diabetes mellitus, dietary assessment

## Abstract

The Food Frequency Questionnaire (FFQ) is one of the most frequently used instruments in epidemiological studies for evaluating dietary intake. Because of the variety of dietary habits within different populations, an FFQ must be tailored to the specific group. To date, no specific FFQ has been developed for type 2 diabetes mellitus (T2DM) patients in Malaysia. In this study, we developed a semi-quantitative FFQ to estimate macronutrient intake among T2DM patients. The development of the FFQ was based on the data acquired from 150 respondents with T2DM from the southern part of Peninsular Malaysia who completed the three-day 24-h dietary recalls. The respondents were selected by convenience sampling. The mean intake from each food item and the proportions of macronutrients were calculated. The approach from a previous study was used to compile a list of foods items with a cumulative 90% macronutrient contribution that is significant for the nutrient of interest. In conclusion, we have successfully developed a new semi-quantitative FFQ with a total of 79 food items and nine food groups. The frequencies of the FFQ were divided into nine categories and this FFQ represents the usual food intake of T2DM patients in Malaysia. However, this tool has yet to be validated in patients with T2DM in Malaysia.

## 1. Introduction

T2DM is a public health issues that has affected 415 million patients globally [1]. Moreover, it is predicted that by 2040 the number of patients with T2DM will increase to 642 million patients worldwide [2]. The prevalence of T2DM showed a significant rise in low- and middle-income countries, including Southeast Asian countries [3]. In Malaysia, the National Health and Morbidity Survey (NHMS) showed that the prevalence of T2DM has increased to 18.3% in 2019 from 17.5% in 2015 [4].

Diabetes mellitus has been linked to microvascular complications such as diabetic nephropathy, retinopathy, and peripheral neuropathy as well as macrovascular conditions, such as coronary heart disease, stroke, and peripheral arterial disease [5,6,7]. Patients with older age, divorce, history of alcohol consumption, poor glycemic control, high body mass index, and longer periods of diabetic duration had higher risk for developing diabetic complications [8,9]. Management and treatment of T2DM patients are significantly influenced by dietary factors [10,11]. Other than that, dietary factors are one of the modifiable risk factors for diabetic complications [12]. For example, a study by Ganesan et al. demonstrated that low dietary fiber intake is associated with diabetic retinopathy. This finding is consistent with a systematic review which demonstrated a reduced caloric intake associated with lower risk of diabetic complication [13].

Diabetes management requires both pharmacological interventions and lifestyle modifications, including dietary management. Therefore, for preventative measures, it is essential to understand the role of dietary intake in chronic diseases [14]. T2DM patients require different dietary and nutritional strategies to ensure good control and prevent diabetic complications [10]. An accurate dietary assessment is required to understand how dietary intake relates to this disease. There are several approaches for measuring nutritional intake globally, including 24-h dietary recalls, dietary records, and FFQs [15]. The information is collected with the assistance of a trained interviewer or by self-reporting [16].

The FFQ is one of the most commonly used methods in epidemiological studies to assess long-term nutritional exposure [17]. FFQs offer a more accurate picture of a dietary pattern and enables researchers to diagnose individuals based on their food intake [18]. Additionally, this tool allows the estimation of long-term dietary intake in a simple method, is easy to understand, time-efficient, minimal cost, and has the ability to show variations in the seasonal intake or occasional food intake [19].

There are a few factors which influence the variation of FFQs such as differences in socioeconomic status, variation in food, geographical or cultural intake patterns, and underlying comorbidity [20,21]. The FFQ should be developed specifically for the purpose of the study, nutrients of interest, or the target population because dietary patterns can be influenced by several factors in the study [22]. People with some medical conditions, such as T2DM, have different dietary behavior compared to people without diabetes [23]. For example, a study found that T2DM patients had lower fiber intake and higher energy intake [24].

Nevertheless, only a few countries namely Australia, Japan, Taiwan, Mali, and Korea have developed and validated FFQs among T2DM patients [25,26,27,28]. Malaysia is made up of a multi-ethnic population with Malays, Chinese, and Indians as the major ethnic groups [29]. Malays constitute more than half of the country’s population, making up the nation’s largest ethnic group. Therefore, the FFQs which have been developed and validated in other countries cannot be used in Malaysia due to the difference in Malaysian dietary intake originating from the diverse and distinctive food cultures from various ethnics residing in the country. Moreover, different countries have their own dietary preferences. For example, the Brazil population consumed traditional foods, such as rice and beans, and consumed a high frequency of ultra-processed foods, such as cookies and carbonated drinks [30]. In the Malaysian population, dietary pattern is more akin to the Asian dietary habit. Hence, the FFQ should represent regional dietary habits and the accuracy of such data needs to take this matter into account. The development of the list of food items is essential to the success of an FFQ since this will reflect the food habits of the target population [20].

To date, there are locally validated FFQs in Malaysia to assess the dietary intake of healthy populations such as pregnant women [31], preschool children [32], primary school-aged children [33], adolescents [34], and adults [35]. There is lack of studies on the development of FFQs among T2DM patients in Malaysia. Previous studies focused on the general and healthy population rather than specific populations and targeting people with T2DM [22,36]. Therefore, the aim of this study is to develop a semi-quantitative FFQ to assess macronutrient intake among T2DM patients in Malaysia.

## 2. Material and Methods

This study is an FFQ development study that involved data analysis using three-day 24-h diet recalls of T2DM patients. Ethical approval was obtained from the Medical Research and Ethics Committee of Universiti Kebangsaan Malaysia (FF-2022-252) and the Medical Research Ethics Committee of the Ministry of Health, Malaysia (NMRR-ID-22-01212-XHN (IIR)). Written informed consent was obtained from the respondents prior to data collection.

During this study, the development of the FFQ was initiated. The development process involved 150 respondents of T2DM patients selected by convenience sampling. This study was conducted among T2DM patients in two districts in Johor, which are located in the southern part of Peninsular Malaysia. The selection of 150 respondents was comparable to a previous study in Brazil in which the development of an FFQ among T2DM patients involved 188 patients, while the study in Korea by Hong et al. involved 85 patients, and the study by Huang et al. involved 126 patients [25,26,37].

To be included in this study, participants had to fulfil the following inclusion criteria: (1) diagnosed with T2DM by a medical doctor, (2) aged between 18 years old and 75 years old, and (3) a Malaysian resident. Exclusion criteria were: (1) diagnosed with debilitating illness such as acute illness, cancer, or activity of daily living dependent, (2) those who refused to consent, or (3) having memory problems. There were no restrictions on gender for this study.

This study was divided into two stages. The first stage involved data collection among respondents of T2DM patients using an interview approach, and the second stage involved data analysis and the creation of an FFQ list from the three-day 24-dietary recall data.

### 2.1. First Stage of the Development Phase

At this stage, all respondents were provided with information regarding the three-day 24-h dietary recalls. Information on food intake from two working days and one non-working day was obtained. The aim of the three-day dietary recall is to provide individual variation in food and dietary intakes [38]. In the dietary recall, the respondents were asked about the foods they eat and how the foods were prepared, for example whether it includes frying, boiling, roasting, steaming, or other methods. The portion size and ingredient used will improve the accuracy of food intake estimation in the FFQ. A flip chart with local food images and household dimensions was used as an aid tool during the interview to help the respondents estimate the size and proportion of the consumed food and beverages.

### 2.2. Second Stage of the Development Phase

**Figure 1:**
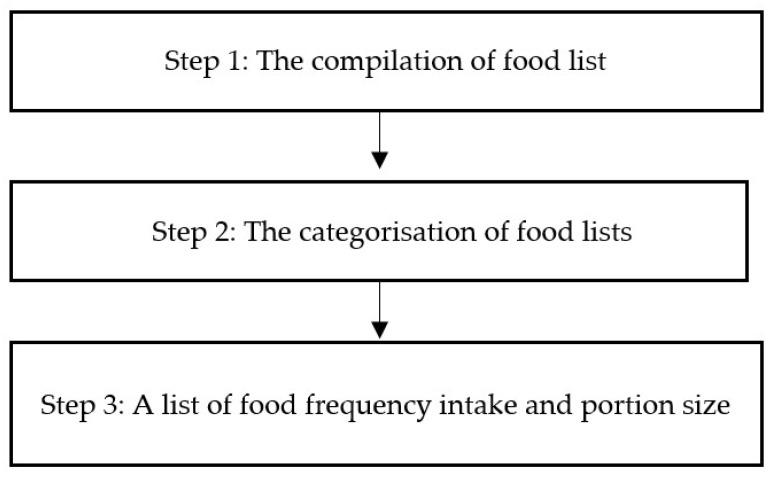
The steps in the second stage of the development phase.

The second stage was further divided into three main steps as shown in Figure 1: (a) the compilation of food lists, (b) the categorization of food lists, and (c) a list of food frequency intake and portion sizes [33]. The food list was derived from the 24-h dietary recall of the respondents. The method used by Tokudome et al. was followed in the process of choosing food items for the FFQ. Consumption of various food items reported by study participants in their three-day 24-h dietary recalls led to the creation of a list of food item codes covering food intake on three days of the week, including two weekdays and one weekend day. All food items from the dietary recall were extracted into an Excel sheet. The food items recorded in the form of household measurements were converted to grams based on the Malaysian Food Composition Database [39] and Atlas of Food Exchanges & Portion Sizes [40].

Total energy and macronutrients intake for each respondent and the nutrients contributed by each food item, as well as the total proportion of energy and macronutrients, were determined using Nutritionist ProTM software (version 7.9.Axxya Systems LLC, Stafford, TX, USA).

Subsequently, the food list was constructed using the approach by Tokudome et al. that includes foods that contributed at least 90% of energy or macronutrients [41,42]. The contribution of intake of each food item was calculated using the formula below [43]:

$$\%\;\mathrm{frequency}\;\mathrm{intake}\;\mathrm{contributed}\;\mathrm{item},\;x=\frac{total\;intake\;of\;specific\;item,\;x}{total\;intake\;of\;all\;food}\times 100.$$

The food items were ranked according to their contribution to energy and macronutrients such as protein, fat, and carbohydrates. The list of the food items and the compilation were based on the Malaysian Food Composition Table [33], foods potentially relevant for the nutrient of interest [21], and the approach formulated by Tokudome et al. which is 90% cumulative macronutrient contribution [20,41].

Then, conceptually, similar foods that share comparable characteristics of nutritional content were aggregated into groups according to the energy, carbohydrates, proteins, and fats content [17,42]. These data were grouped into general food groups that were appropriate for the FFQ, according to methods by Block et al. [42]. For example, two rice types (boiled and fried, etc.) were all assigned a single code, “cereal and cereal products”. In the merging process, each food item was further grouped together with similar foods. Various cooking methods for a single food item were counted as one food item. For example, curried chicken and fried chicken were classified into “chicken”.

Total calories, carbohydrates, proteins, and fats were all included in the food database. The Malaysian Food Composition Table, Malaysian Food Nutrient Composition and Atlas of Food Exchanges & Portion Sizes [40], Malaysians Food Composition Database [39], and food labels were used to obtain nutrient information and to estimate the nutrient content of the raw ingredients reported in the recipes.

When there were no data available from any database for specific food products, a standard recipe was created and the nutrient information was completed using Nutritionist ProTM (Axxya Systems LLC, Stafford, TX, USA) software. Once the list of the food items was compiled, the frequency of consumption and the portion size of each food item was constructed [22].

### 2.3. Pre-Test of the FFQ

The newly developed FFQ was pre-tested among ten T2DM patients, and they were invited to provide their feedback. They were given an evaluation form that consists of: (a) the identity or familiarity of the food items; (b) the clarity of the questionnaire (layout, color, font, and size); (c) portion size; (d) relevance to dietary practice; and (e) the flow of the FFQ with these items rated on a 5-point Likert scale (1 = very poor, 2 = poor, 3 = fair, 4 = good, and 5 = very good). A panel of experts consisting of a nutritionist and dietitian also reviewed the semi-quantitative FFQ on the instructions, questionnaire layouts, portion size estimate, and food item list to ensure its appropriateness and to help increase the quality of the content. Based on this assessment, the FFQ was further revised. Our newly developed semi-quantitative FFQ was in paper format.

### 2.4. Data Analysis

All statistical analyses were performed with SPSS (version 25.0 (SPSS, Inc., Chicago, IL, USA)). Descriptive statistics were expressed as mean and standard deviations (SD) for continuous data. Categorical data were described as frequency (*n*) and percentage (%).

## 3. Results

### 3.1. First Stage of the Development Phase

The FFQ development phase involved 150 T2DM patients. The demographic characteristics of the respondents are shown in Table 1. The mean age of the respondents was 52.30 (SD 6.70) years. The study respondents were predominantly female (56.7%). Most of the respondents were Malay (66.7%), followed by Chinese (20.0%), and Indian (13.3%). A total of 43.3% of the respondents were obese and 33.3% were overweight. The collection of three-day 24-h diet recalls yielded 450 recalls from a total of 150 respondents and the information was utilized for the development of the FFQ.

### 3.2. Second Stage of the Development Phase

The average total energy, carbohydrates, protein, and total fats were calculated. Table 2 shows the mean (SD) of daily energy intake in the FFQ development phase, which was 1610.60 (SD 358.32) kcal. For carbohydrate, protein, and fat, the values were 281.52 (SD 31.54) g, 52.70 (SD 24.74) g and 35.32 (SD 16.15) g, respectively.

All food items and mixed dishes obtained using the 24-h diet recall were collected and divided into nine food groups [42] namely, “cereals and cereal products”, “meat and poultry”, “fish and seafood”, “egg and egg dishes”, “vegetables, “fruits”, “traditional Malaysian kuih”, “milk and milk products”, and “drink and beverages”.

In the end, this phase yielded a total of 75 food items. Foods that did not provide any of the essential nutrients were excluded [44]. The food items that contributed up to 90% of total energy intake, carbohydrates, protein, and fat are presented in Table 3. Eight food items contributed to 90% of total energy intake, nine food items for carbohydrates, seven food items for protein, and eight food items for fats. After the creation and categorization of the food lists, the next steps involved calculating the frequency of the newly developed FFQ and determining the portion sizes.

In our study, the frequency of the FFQ was categorized into nine categories. The portion size in our FFQ was based on household measurement items including bowl, plate, and tablespoon.

## 4. Discussion

This study describes the process of semi-quantitative FFQ development for T2DM patients in Malaysia. The development of the semi-quantitative FFQ involved 150 T2DM patients who are predominantly Malay, followed by Chinese and Indian ethnicities. Malaysia is a multiracial country with a variety of dietary intakes due to differences in race and cultural practices [45,46]. This is the first FFQ developed for T2DM patients in Malaysia. Our FFQ took into account the foods that are consumed the most frequently by T2DM patients, and it accurately reflects the dietary practices that are common in Malaysia.

Based on the findings, cereal and cereal products such as noodles, roti canai, white rice, and chicken were the main contributors to total energy intake in our FFQ, which contributed up to 90% of the total energy intake (Table 2). This food group was considered a staple food by the Malaysian population. Rice is a staple food in Malaysia, specifically to the Malays, accounting for the majority of the carbohydrate source [47,48]. The major contributors to the total protein intake were chicken, meat, and fish, which contributed 61.90% of the total protein intake among T2DM patients. For fat, chicken, fish, and milk were the major contributors which account for 54.73% of the total fat intake.

This list of food items is crucial to the effectiveness of an FFQ because it should reflect the dietary preferences of the target population. In comparison to the validated FFQ in the study by Huang et al. [25] for T2DM in Japan, which consists of 45 food items and 85 food items and 12 food groups in the validated FFQ for Korean T2DM patients [26], our semi-quantitative FFQ included a total of 79 food items and nine food groups as shown in Table 4, and consisted of the foods most commonly consumed by T2DM patients. Cade et al. [20] mentioned that the range of food items in the FFQ was between 5–350 items. Therefore, based on previous research, the total number of items in our newly developed FFQ is appropriate, as food items less than 50 items may underestimate food intake, and an FFQ with more than 100 items may overestimate food intake and burden the respondents [49].

The nine food groups in our FFQ were “cereals and cereal products”, “meat and poultry”, “fish and seafood”, “egg and egg dishes”, “vegetables”, “fruits”, “traditional Malaysian kuih”, “milk and milk products”, and “drink and beverages”. The nine food groups, which were categorized using the Atlas of Food Exchanges & Portion Sizes, consist of the food items that contributed up to 90% of energy and the macronutrient [40]. The ethnic diversity within Malaysian communities influences the dietary pattern among T2DM patients in Malaysia, as can be seen from the varieties of their dietary consumption [46]. For example, there are various types of cereal and cereal products widely available and highly influenced by the cultural and tradition practices in the Malaysian population, such as white rice, fried rice, rice cooked with coconut milk, plain porridge, fried noodles, noodles with gravy, roti canai, chapati, and tosai.

In our findings, Malay respondents preferred rice cooked with coconut milk, also called “nasi lemak“, or roti canai for breakfast, whereas Indian respondents preferred chapati or tosai for breakfast. In Malaysia, nasi lemak is one of the most well-known rice-based breakfasts, while traditional “kuih” are consumed for breakfast or evening tea [50].

Furthermore, as shown in Table 4, our FFQ consists of traditional Malaysian “kuih” such as banana fritters, curry puff, cucur udang, sri muka, lopes, and kuih lapis which are food items related to our Malaysian cultural and tradition [48]. The traditional Malaysian “kuih” contributed 9.47% for energy, 4.88% for carbohydrates and 8.52% for protein. The Malaysian Adult Nutrition Survey (MANS) reported that local “kuih” are one of the top ten weekly consumed foods. It was found that 16.3% of Malaysian adults consumed about two pieces of local “kuih” daily, while 63.5% of Malaysian adults consumed at least four pieces of local “kuih” weekly [51].

Other than the list of food items, the frequency response section and portion size of the food are the key components in the design and development of our FFQ. In terms of food frequency, we used nine categories of frequency as these were the most common categories in the previous FFQ [20]. The nine categories are (a) never or less than once a month, (b) one to three per month, (c) one per week, (d) two to four per week, (e) five to six per week, (f) one per day, (g) two to three per day, (h) four to five per day, and (i) six or more per day [20]. Based on these nine categories, only one choice can be selected for each food item. Our FFQ will use food categories rather than exact frequencies to avoid minimal loss of information during estimates of food intake. Bowl, plate, and tablespoon were used as the portion size for each item in our FFQ according to The Nutrient Composition of Malaysian Foods and the Atlas of Food Exchanges & Portion Sizes [40].

The next step was to conduct a validation study. Other studies have recommended that the respondents of the development phase should be excluded from the validation study [33].The strengths of this study include a comprehensive food items list derived from a survey-specific population of T2DM patients in Malaysia. Furthermore, we used a local nutrient database and food composition tables during the development of the FFQ. In our study, we used three-day 24-h dietary recalls, which have been suggested to capture some individual variation in food and nutrient intakes. 

There are some limitations noted in this study. This study only discussed the development phase and preliminary testing. The newly developed FFQ will be validated with 100 T2DM patients. This validation process aims to ensure the newly developed FFQ is valid and accurate. The calculation of the total sample size based on Pritchard et al. 2010 states that the sample size for the validation phase of the FFQ among patients with T2DM is 50 to 100 respondents [52]. In that phase, the food intake from the new FFQ will be compared with three days of 24-h dietary recall. Then, the reproducibility study will be conducted on a subsample of 30 subjects who participated in the validation phase through the repeat administration of the FFQ, two weeks after the first administration of the FFQ. Other than that, there was recall bias when receiving the information for the three-day 24-h dietary recalls and the possibilities of underestimation and overestimation of dietary intake. However, this limitation was reduced by using food photos and standard household measurements.

## 5. Conclusions

In conclusion, we developed a new FFQ with 79 food items and nine food groups for T2DM patients. The development of an FFQ among T2DM patients in this study is useful and crucial in estimating dietary intake and evaluating the relationship between diet and diabetes complications among T2DM patients in the Malaysian population. However, further validation is needed for this newly developed FFQ.

## Figures and Tables

**Table 1:** Demographic characteristics of the respondents (*n* = 150).

Demographic Characteristics	*n* (%)	Mean (SD)
Age (Years)		52.30 (6.70)
Gender		
Male	65 (43.3)	
Female	85 (56.7)	
Ethnicity		
Malay	100 (66.7)	
Chinese	30 (20.0)	
Indian	20 (13.3)	
Body Mass Index (BMI) *		
Normal	35 (23.3)	
Overweight	50 (33.3)	
Obese	65 (43.4)	

* Based on WHO BMI cut-off points for Asian population.

**Table 2:** Nutrient intake of the respondents from the FFQ development phase (*n* = 150).

Parameters	Mean (SD)
Total energy (kcal/day)	1610.60 (358.32)
Carbohydrates (g/day)	281.52 (31.54)
Protein (g)	52.70 (24.74)
Total fat (g)	35.32 (16.15)

**Table 3:** Percentage contribution and cumulative percentage contribution of the foods for energy, carbohydrates, protein, and fat.

Variable	No. *	Food Item	Percentage Contribution to Energy or Macronutrient Intake (%)	Cumulative Percentage (%)
Energy	1	Noodles	17.45	17.45
	2	Roti canai	16.51	33.96
	3	White Rice	14.55	48.51
	4	Chicken	13.59	62.10
	5	Milk	10.12	72.22
	6	Kuih-muih	9.43	81.65
	7	Egg	5.22	86.87
	8	White bread	4.60	91.47
Carbohydrate	1	White Rice	21.70	21.70
	2	Roti canai	16.94	38.64
	3	Noodles	12.77	51.41
	4	Biscuit	10.29	61.70
	5	White bread	8.90	70.60
	6	Meats	6.00	76.60
	7	Beverages	5.32	81.92
	8	Kuih muih	4.88	86.80
	9	Fruits	3.42	90.22
Protein	1	Chicken	24.76	24.76
	2	Meats	18.78	45.54
	3	Fish	16.36	61.90
	4	Noodles	11.94	73.84
	5	Egg	7.73	81.57
	6	White Rice	7.59	89.16
	7	White Bread	5.31	94.47
Fat	1	Chicken	20.71	20.71
	2	Fish	19.82	40.53
	3	Milk	14.20	54.73
	4	Egg	14.15	68.88
	5	Kuih-muih	8.52	77.40
	6	Biscuit	7.67	85.07
	7	Noodles	4.28	89.35
	8	Meats	2.86	92.21

* “No.” is the abbreviation of “Number” which refers to the ranking of food items according to their percentage of energy and macronutrient contributions.

**Table 4:** Description of food group and food items in the FFQ.

Food Group	Food Items
Cereal and cereal products	White rice, fried rice, rice cooked with coconut milk, plain porridge, fried noodles, noodles with gravy, roti canai, chapati, tosai, white bread, wholemeal bread, bun, biscuit creams, oat
Meat and poultry	Fried chicken, chicken dishes cooked with coconut milk, cooked in soup, meat cooked in soup, roasted chicken, beef, mutton,
Fish and seafood	Fried fish, fish cooked with coconut milk, fish cooked without coconut milk, seafood
Egg and egg dishes	Boiled egg, fried egg, salted egg
Vegetables	Fried green vegetables, mustard, spinach, swamp cabbage, bean, cucumber, yam, pea, carrots, tomato, salad, corn, mushroom
Fruits	Banana, guava, papaya, apples (red and green), watermelon
Traditional Malaysian “ kuih”	Banana fritters, curry puff, cekodok pisang, cucur udang, sri muka, lopes, kuih lapis
Milk and milk products	Fresh milk, milk powder, krimer, sweetened milk
Drink and beverages	Teh O, Teh tarik (with milk), coffee ‘O’ (with sugar), coffee with sugar, malt drink, cordial drink, fruit juice

## Data Availability

The data presented in this study are available in this article.
